# miR-497-5p Decreased Expression Associated with High-Risk Endometrial Cancer

**DOI:** 10.3390/ijms22010127

**Published:** 2020-12-24

**Authors:** Ivana Fridrichova, Lenka Kalinkova, Miloslav Karhanek, Bozena Smolkova, Katarina Machalekova, Lenka Wachsmannova, Nataliia Nikolaieva, Karol Kajo

**Affiliations:** 1 Department of Genetics, Cancer Research Institute, Biomedical Research Center of Slovak Academy of Sciences, 84505 Bratislava, Slovakia; Lenka.Kalinkova@savba.sk (L.K.); Lenka.Wachsmannova@savba.sk (L.W.); Nataliia.Nikolaieva@savba.sk (N.N.); 2 Laboratory of Bioinformatics, Biomedical Research Center of Slovak Academy of Sciences, 84505 Bratislava, Slovakia; Miloslav.Karhanek@savba.sk; 3 Department of Molecular Oncology, Cancer Research Institute, Biomedical Research Center of Slovak Academy of Sciences, 84505 Bratislava, Slovakia; Bozena.Smolkova@savba.sk; 4 Department of Pathology, St. Elisabeth Cancer Institute, 81250 Bratislava, Slovakia; katarina.machalekova@ousa.sk (K.M.); karol.kajo@ousa.sk (K.K.)

**Keywords:** miR-497-5p expression, endometrioid endometrial carcinoma, serous endometrial carcinoma, rare subtypes of endometrial carcinoma, machine learning evaluation

## Abstract

The current guidelines for diagnosis, prognosis, and treatment of endometrial cancer (EC), based on clinicopathological factors, are insufficient for numerous reasons; therefore, we investigated the relevance of miRNA expression profiles for the discrimination of different EC subtypes. Among the miRNAs previously predicted to allow distinguishing of endometrioid ECs (EECs) according to different grades (G) and from serous subtypes (SECs), we verified the utility of miR-497-5p. In ECs, we observed downregulated miR-497-5p levels that were significantly decreased in SECs, clear cell carcinomas (CCCs), and carcinosarcomas (CaSas) compared to EECs, thereby distinguishing EEC from SEC and rare EC subtypes. Significantly reduced miR-497-5p expression was found in high-grade ECs (EEC G3, SEC, CaSa, and CCC) compared to low-grade carcinomas (EEC G1 and mucinous carcinoma) and ECs classified as being in advanced FIGO (International Federation of Gynecology and Obstetrics) stages, that is, with loco-regional and distant spread compared to cancers located only in the uterus. Based on immunohistochemical features, lower miR-497-5p levels were observed in hormone-receptor-negative, p53-positive, and highly Ki-67-expressing ECs. Using a machine learning method, we showed that consideration of miR-497-5p expression, in addition to the traditional clinical and histopathologic parameters, slightly improves the prediction accuracy of EC diagnosis. Our results demonstrate that changes in miR-497-5p expression influence endometrial tumorigenesis and its evaluation may contribute to more precise diagnoses.

## 1. Introduction

Endometrial cancer (EC) is one of the most prevalent gynecological malignancies in the world [1]. It is the sixth most common malignancy in women, with over 380,000 new cases and almost 90,000 deaths in women worldwide in 2018 [2].

The incidence of EC is rapidly increasing worldwide, with the highest disease burden reported in North America and Western Europe. The number of new cases and deaths is projected to increase in 2025, by 20.3% and 17.4%, respectively, compared to 2018 [2]. These negative trends reflect both the aging of populations and the rise in obesity [3].

EC has generally been divided into two clinicopathological categories. The first group, classified as type I, represents the majority of cases (80–90%) and is associated with a hyperplastic and estrogen-related endometrium and endometrioid types of carcinoma (EEC). It occurs primarily in obese pre-, peri-, and early postmenopausal women and has a good prognosis. Type II is characterized by a non-estrogenic and atrophic endometrium and high-grade carcinomas, such as serous carcinoma (SEC), clear cell carcinoma (CCC), and carcinosarcoma (CaSa). It affects mostly postmenopausal women and is associated with a high risk of relapse and metastasis [4,5]. While this classification system has been used since the 1980s, increasing evidence suggests that it is imperfect [6].

The current guidelines for diagnosis and treatment of EC are based on the clinicopathologic factors age, FIGO (International Federation of Gynecology and Obstetrics) stage, histologic type and grade, myometrial invasion, and the presence of lymphovascular space invasion (LVSI), but do not include molecular alterations [7]. Microscopy-based diagnoses are the current standard and serve as important background for (adjuvant) treatment decisions. However, considerable interobserver variations, mostly in high-grade EC, have been recognized, and a centralized review prior to trial inclusion pointed out that the therapeutic consequences are not negligible [8]. Furthermore, the current clinicopathological approach is often insufficient for prognosis. Therefore, the inclusion of additional criteria such as biological expression profiling will be needed to allow both effective surgical management and adjuvant therapy [9]. Of the many advances in the field of EC during the last decade, the one with perhaps the greatest impact is the molecular classification proposed by The Cancer Genome Atlas (TCGA) [10], which has revealed important heterogeneity in tumors with otherwise comparable histological type and grade.

Another promising and prospective field of research in EC is the evaluation of epigenetic regulation involving the protein non-coding RNA molecules microRNAs (miRNAs). Several studies suggest that miRNAs are dysregulated in ECs. They impact the development and progression of ECs and could be helpful in the effective clinical management of the disease [11]. Several studies have shown that the miRNAs expression profiles appear to be associated with prognostic factors such as lymph node involvement [12], LVSI [13], overall survival [12], and recurrence-free survival [9,12]. Knowledge of the expression profile of miRNAs would likely explain certain molecular mechanisms associated with EC and could serve as a basis for diagnostic tests, prognosis, or the identification of potential novel therapeutic targets [9].

With the emergence of new technologies in every sphere including medicine, the large amount of mixed cancer data, such as clinical and genomic data, can be collected and used by the research community for analysis or prediction. Specifically, new approaches related to the profiling of miRNAs have been shown to be a promising tool in cancer detection and identification [14]. Machine learning (ML), an effective data mining tool, can be used in solving problems that are too complex for traditional approaches or that lack known algorithms. Advantages of ML include the flexibility and scalability of the system compared to traditional biostatistical methods, and that it can be applied for risk stratification, diagnosis and classification, and survival prediction [15].

In a previous study, we analyzed 84 miRNAs in endometrial tumor samples of EEC grade 1 (G1) and grade 3 (G3) subtypes and SECs in 20, 21, and 21 patients, respectively. We identified let-7c-5p, miR-125b-5p, miR-23b-3p, and miR-99a-5p as allowing the discrimination of different grades in endometrioid subtype based on expression evaluation, and let-7g-5p, miR-195-5p, miR-34a-5p, and miR-497-5p in the case of distinguishing endometrioid and serous carcinomas [16]. In this study, we verified miR-497-5p from among the miRNAs previously predicted to allow the distinguishing of EECs according to different grades and from SECs. We observed more significant downregulation of miR-497-5p expression in non-endometrioid EC subtypes, namely in SECs, CCCs, and CaSas, compared to EECs. Furthermore, we found significantly decreased miR-497-5p levels in EC groups with high-grade (EEC G3, SEC, CaSa and CCC) compared to low-grade carcinomas (EEC G1 and mucinous carcinoma, MC) and ECs with advanced FIGO stages. The evaluation of immunohistochemical features demonstrated considerable reduction in the miR-497-5p levels of hormone-receptor-negative, p53-positive, and highly Ki-67-expressing ECs. Using ML, we observed approximately 60–80% probability of correct diagnosis in the main EC subtypes when considering only traditional clinical and histopathologic parameters, and the inclusion of miR-497-5p expression results improved the prediction accuracy of EC diagnosis.

## 2. Results

**Figure 1:**
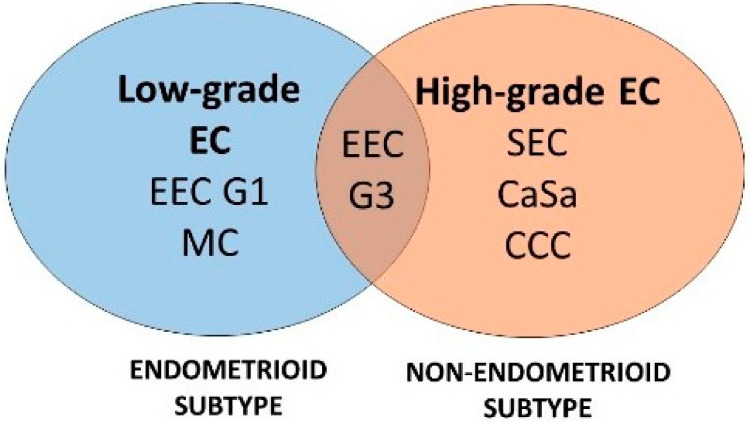
Study diagram of evaluated subtypes of endometrial carcinoma. Numbers of evaluated EC samples were 25, 26, 23, 21, 20, and 5 for EEC G1, EEC G3, SEC, CaSa, CCC, and MC, respec Table 1. and G3, grade 1 and grade 3.

In the present study, we verified the discriminating ability of previously identified miRNAs based on expression levels in an independent group of 120 EC patients. The evaluated EC subtypes are displayed in Figure 1. Then, in all 182 patients (62 from previous study and 120 new patients), the associations between miRNA expression and clinicohistopathological and immunohistochemical data were confirmed, together with an evaluation of the clinical relevance of miRNA expression profiles for EC classification.

### 2.1. Verification of Discriminating miRNAs

We investigated the expression profiles of 11 miRNAs, 8 discriminating miRNAs and 3 miRNAs that were previously downregulated in more aggressive subtypes, but did not have significant differences compared to early ECs, namely miR-145-5p in carcinomas with high-grade compared to low-grade EECs and miR-143-3p and miR-424-5p in the serous compared to endometrioid subtypes. The analyses were performed in other independent groups of EEC G1 or G3 and serous cancer in 25, 26, and 23 patients, respectively. The normalized expression levels of let-7c-5p, miR-125b-5p, miR-23b-3p, miR-99a-5p, miR-145-5p, let-7g-5p, miR-195-5p, miR-34a-5p, miR-497-5p, miR-143-3p, and miR-424-5p in the non-neoplastic endometrium (1.455, 16.846, 5.279, 3.301, 9.490, 1.260, 3.421, 0.593, 0.552, 4.544, and 2.586, respectively) were obtained from a previous study [16].

The expression analyses of miRNAs that were previously identified for discriminating EECs with different grades (Table S1) showed a downregulation (with fold changes less than 0.5) of miR-125b-5p, miR-23b-3p, miR-99a-5p, and miR-145-5p in both EEC G1 and G3 compared to non-neoplastic endometrium and with significant differences between EC subtypes and controls. let-7c-5p was downregulated only in cancers with G3. However, there was no downregulation nor any significant differences between EEC G1 and G3 in terms of expression of the five evaluated miRNAs (Table 1).

For miRNAs previously identified for discriminating EEC from SEC (Table S1), miR-195-5p, miR-143-3p, and miR-424-5p were downregulated in both cases and were significantly different compared to control tissues. Expression of miR-34a-5p was downregulated only in SEC. When comparing expression of miRNAs in SEC to EEC, let-7g-5p, miR-143-3p, and miR-424-5p were downregulated in SEC (Table 1). In this study, only miR-497-5p was downregulated in EEC (fold change 0.256) and SEC (fold change 0.081) compared to controls, together with being downregulated in SEC compared to the EEC (fold change 0.318). Significant differences in miR-497-5p expression in both EEC and SEC compared to control and also between SEC and EEC were found (*p* < 0.001 in all three comparisons) (Table 1). The normalized miR-497-5p relative expression levels for EEC, SEC, and non-neoplastic endometrial tissues represented by 2^−ΔΔCt^ values were 0.205, 0.059, and 0.937, respectively.

Considering the interindividual variability in the molecular characteristics of ECs based on previous results, we identified three significantly downregulated miRNAs in SEC compared to EEC, but only miR-497-5p was verified to allow discrimination of these two subtypes in an independent group of EC patients. In this study, miR-143-3p and miR-424-5p were downregulated in both subtypes compared to controls, and also in SEC compared to EECs, but in contrast to previous negative results, significant differences between expressions of evaluated EC groups were observed; therefore, more detailed investigation of these miRNAs is necessary.

### 2.2. miRNA Expression in Rare Subtypes of Endometrial Cancer

**Figure 2:**
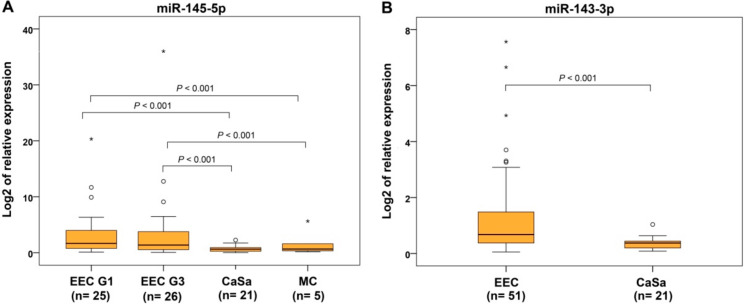
Relative miRNA expression in endometrioid, serous, and rare subtypes of endometrial carcinoma. Differential expression of miR-145-5p of endometrioid endometrial carcinomas grade 1 (EEC G1) and grade 3 (EEC G3) compared to carcinosarcomas (CaSa) and mucinous endometrial carcinoma (MC) (**A**) and miR-143-3p differential expression between EECs and CaSas (**B**). The lengths of the boxes show the interquartile range (IQR), which represents values between the 75th and 25th percentiles. (○) labels outliers (values more than 1.5 IQRs and less than 3 IQRs from the end of the box). (*) represents values greater than three IQRs. The horizontal line depicts the median. Statistical significance is regarded as *p* < 0.05.

In addition to 51 EECs and 23 SECs, we also quantified miRNA expressions in rare endometrial subtypes, namely 21 CaSas, 20 CCCs, and 5 MCs. From 11 evaluated miRNAs, the expressions of 9 were downregulated in CaSa subtype, with statistically significant differences found between CaSa and control, with let-7c-5p and let-7g-5p being the exceptions. In the CCCs, downregulation was observed for all miRNAs except let-7g-5p and for MCs, in seven miRNAs compared to controls (Table 2 and Table 3). In our results, several examples allowing discrimination between endometrioid and rare subtypes were evidenced. Based on significant differences in expression levels, miR-145-5p could distinguish CaSa and MC from EEC subtypes regardless of tumor grade (Table 2, Figure 2A), and miR-143-3p allowed distinguishing CaSas from EECs (Table 3, Figure 2B). Finally, the ability of miR-497-5p to discriminate CaSas (fold change 0.467, *p* = 0.005) and CCCs (fold change 0.454, *p* = 0.008) from EECs but not from SEC was verified (Table 3), which indicates the greater similarity of these aggressive rare EC subtypes and SECs in terms of molecular features.

### 2.3. miR-497-5p Expression and Clinicohistopathological Characteristics

For evaluation of the associations between miR-497-5p expression and clinical features and histopathological characteristics (Table 4), we utilized data from 62 and 120 EC patients from the previous and present study, respectively.

**Figure 3:**
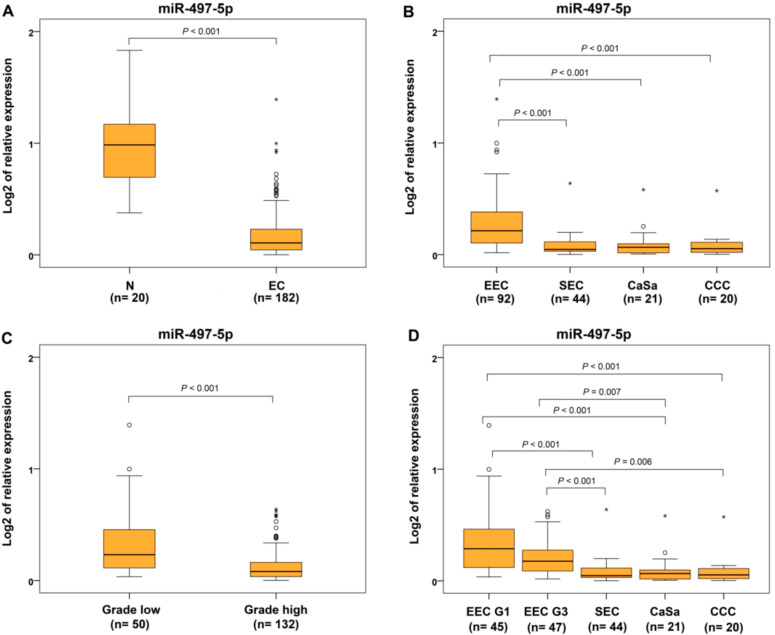
Relative expression of miR-497-5p in different subtypes of endometrial carcinomas and controls. Differential expression between non-neoplastic endometrium (N) and endometrial carcinoma (EC) (**A**), endometrioid (EEC) or, separately, EEC G1 and G3, and serous (SEC) endometrial carcinomas, carcinosarcomas (CaSa), clear cell carcinomas (CCC) (**B**,**D**), low-grade (EEC G1, MC), and high-grade (EEC G3, SEC, CaSa, CCC) subtypes (**C**). The lengths of the boxes show the interquartile range (IQR), which represents values between the 75th and 25th percentiles. (○) labels outliers (values more than 1.5 IQRs and less than 3 IQRs from the end of the box). (*) represents values greater than three IQRs. The horizontal line depicted median. Statistical significance is regarded as *p* < 0.05.

In this group of patients, the statistical difference in miR-497-5p expression and decreased levels were validated in all ECs compared to controls (Figure 3A) and in SEC, CaSa, and CCC subtypes compared to EECs as well as compared to EEC G1 and EEC G3 sub-categories (Figure 3B,D). Comparing miR-497-5p expression in EC groups of different grades, reduced expression was found in high-grade cases (EEC G3, SEC, CaSa and CCC) compared to low-grade carcinomas (EEC G1 and MC) (Figure 3C).

**Figure 4:**
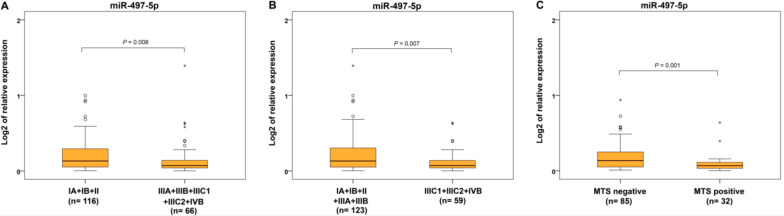
Relative expression of miR-497-5p in different International Federation of Gynecology and Obstetrics (FIGO) stages and metastasizing and non-metastasizing endometrial carcinomas. Differential expression between grouped FIGO categories: carcinomas in uterus (IA, IB, and II) and with local or regional involvement with lymph node and distant metastases (IIIA, IIIB, IIIC1, IIIC2, and IVB) (**A**), carcinomas in uterus or locally invading without metastases (IA+IB+II+IIIA+IIIB) and those including lymph node and distant metastases (IIIC1+IIIC2+IVB) (**B**), patients without and with distant metastases (MTS-negative and MTS-positive) (**C**). The lengths of the boxes show the interquartile range (IQR), which represents values between the 75th and 25th percentiles. (○) labels outliers (values more than 1.5 IQRs and less than 3 IQRs from the end of the box). (*) represents values greater than three IQRs. The horizontal line depicted median. Statistical significance is regarded as *p* < 0.05.

In terms of FIGO categories, we found significantly decreased miR-497-5p expression in advanced cases of EC with locoregional involvement and distant metastases (IIIA, IIIB, IIIC1, IIIC2, and IVB) compared to cancers located only in the uterus (IA, IB, and II) (Figure 4A). Significantly reduced levels were seen in the group of EC with lymph node and distant metastases (IIIC1+IIIC2+IVB) compared to carcinomas located only in the uterus or invading only locally without metastases (IA+IB+II+IIIA+IIIB) (Figure 4B). When the presence of metastatic lymph nodes and distal metastases were separately evaluated, decreased levels of miR-497-5p were found in ECs with metastases (Figure 4C) but not in cancers with positive lymph nodes. Similarly, no statistical differences in miR-497-5p expression were observed in comparison of T1b vs. T1a and T3 vs. T1 and T2.

**Figure 5:**
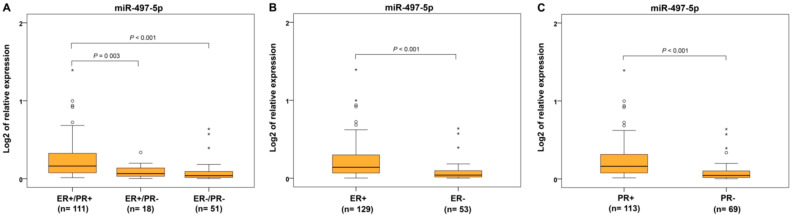
Relative expression of miR-497-5p in endometrial carcinomas with different hormonal receptor status. Differential expression between endometrial carcinomas grouped according to estrogen (ER) and progesterone (PR) receptor expressions, namely ER+/PR+, ER+/PR−, and ER−/PR− (**A**); carcinomas with estrogen receptor expression in 1% and >1% of positively responding cells (ER+ and ER−) (**B**); carcinomas with progesterone receptor expression in 1% and >1% of positively responding cells (PR+ and PR−) (**C**). The length of the boxes is the interquartile range (IQR) that represents values between the 75th and 25th percentiles. (○) labels outliers (values greater than 1.5 IQRs and less than 3 IQRs from the end of the box). (*) represents values greater than three IQRs. The horizontal line depicts the median. Statistical significance is regarded as *p* < 0.05.

**Figure 6:**
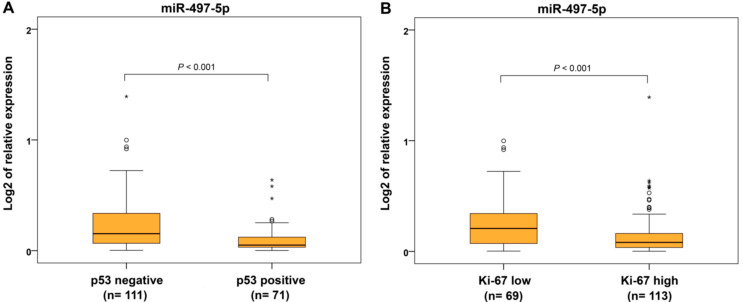
Relative expression of miR-497-5p in endometrial carcinomas with different immunohistochemical parameters. Differential expression between p53-negative (<90% of responsive cells) and p53-positive (≥90% of responsive cells or showed complete absence) endometrial carcinomas (**A**), and carcinomas with proliferative index Ki-67-low (≤50% of responsive cells) and Ki-67-high (˃50% of responsive cells) (**B**). The lengths of the boxes show the interquartile range (IQR), which represents values between the 75th and 25th percentiles. (○) labels outliers (values more than 1.5 IQRs and less than 3 IQRs from the end of the box). (*) represents values greater than three IQRs. The horizontal line depicted median. Statistical significance is regarded as *p* < 0.05.

From the immunohistochemical parameters, we evaluated protein expression of estrogen and progesterone receptors (ER and PR), p53 and Ki-67 proliferative factor. Expression of miR-497-5p was significantly decreased in ER−/PR− and ER+/PR− tumors compared to ER+/PR+ carcinomas (Figure 5A). For separately estimated categories of hormonal receptors, ER− and PR− cancers showed decreased miR-497-5p levels compared to ER+ and PR+ ECs, respectively (Figure 5B,C). Identical results were found in p53-positive and highly Ki-67-expressing cancers compared to those with p53-negative and low Ki-67 expression, respectively (Figure 6A,B).

The association with selected clinicopathological parameters indicates decreased miR-497-5p expression in more aggressive high-grade EC subtypes such as EEC G3, SEC, CCC, and CaSa, as well as in cases of EC in advanced FIGO stages and in patients with metastatic propagation and hormone-receptor-negative, p53-positive, and highly Ki-67-expressing tumors.

### 2.4. Machine Learning Analyses

The ML analyses were performed in several scenarios using data from all 182 patients, including or excluding some clinical parameters represented here as input features and selecting classification types as target variables. Firstly, a comparative evaluation was done of the performance on subsamples of EEC and SEC subtypes and the three data mining algorithms, SVM (support vector machine), Neural Network, and Random Forest, rated according to their classification accuracy (CA), precision rate, area under ROC curve (AUC), F1 score, and recall (Table S4). All three classification methods presented only small differences between AUC, CA, F1, precision, and recall parameters. The lift curves for the data mining analyses for the six target subtypes showed variations in the predictive capabilities of the three data mining algorithms, and SVM and Neural Network presented the same performance for EEC G1 followed by EEC G3, SEC, and CCC subtypes, and CaSa and MC showed the worst performance (Figure S1). For estimation of predictions for EC diagnosis, we chose the SVM method, mainly because of fewer false-positive predictions for some subtypes such as EEC G3 (Table S5).

Confusion matrix performance analyses for SVM were performed with exclusion and inclusion of miR-497-5p expression results according to different scenarios with respect to the data characteristics, namely discrete, continuous, or mixed data. Our results show that involving the mixed or discrete immunohistochemical characteristics of ECs resulted in better performance of ML for most of the target EC subtypes compared to those with discrete characteristics including T, FIGO, and grade distribution (Table 5).

Analyses of the data on clinical and histopathological features combined with miR-497-5p expression results led to the improved prediction of EEC G1, EEC G3, and SEC subtypes by 2.2%, 6.3%, and 6.8%, respectively, using the discrete values of the immunohistochemical results. Continuous values of ER%, PR%, Ki-67%, and age improved predictions of ECC G1, EEC G3, and CaSa by 2.2%, 4.3%, and 4.7%, respectively, and a mix of all evaluated characteristics increased the correct prediction of EEC G1, EEC G3, and SEC by 4.4%, 2.1%, and 4.6%, respectively. On the other hand, miR-497-5p impaired predictions for EEC G3, SEC, CaSa, and CCC by 2.1–14.3% when the discrete features of ER, PR, Ki-67, p53, T, FIGO, and grade were used. These results show the sensitive nature of utilizing miR-497-5p and selecting discrete or continuous input features. Involvement of only discrete input features showed that they are more crucial than miR-497-5p expression results, especially clinical data such as T, FIGO, and grade, the detection of which strongly depends on the pathologist’s expertise. However, using less expertise-dependent input features, such as ER, PR, Ki-67, and p53, or their continuous values together with miR-497-5p, gives improved prediction results without a strong dependence on the pathologist’s expertise.

In accordance with lift curves (Figure S1) and confusion matrix tables (Table 5), it was observed that the ML algorithms were better able to predict the EEC G1, EEC G3, and SEC than CaSa and CCC subtypes. The main reason for undefined predictions for the MC subtype could be that only five MC samples were available in this study.

## 3. Discussion

Among the previously identified miRNAs, which discriminate G1 and G3 in EECs and endometrioid from serous ECs, only miR-497-5p was verified in the independent group of ECs. Results from experiments in cervical, renal, non-small-cell lung, osteosarcoma, and hepatocellular cancer cells show that miR-497-5p regulates cell growth and proliferation and is associated with the MAPK pathway by targeting several involved genes such as *RAF1*, *KDR*/*VGFR-2*, and *IGF1-R* [17,18,19,20,21]. Similarly, in a colorectal cancer sample, it was shown that miR-497-5p inhibits tumor growth by targeting the *IRS1* gene that influences several signaling pathways, including MAPK [22]. Our recent study also identified the involvement of miR-497-5p in MAPK signaling in EC samples [16]. Another cervical cancer study showed that miR-497-5p inhibits cell proliferation through *CBX4* targeting, which resulted in cell cycle arrest at the S phase and decreased expression of CDK2 and cyclin A2 proteins [23]. Furthermore, miR-497-5p modulates cell proliferation, invasion, and survival through the epithelial-to-mesenchymal transition (EMT) regulation by targeting *SERPINE-1* and *SLUG/SNAI2* genes [24,25,26]. In renal cancers, miR-497-5p inhibits cell proliferation by targeting the *PD-L1* gene, which contributes to promoting apoptosis [27]. In breast cancer cells, the direct regulation of miR-497-5p transcription by ER-alpha was experimentally confirmed, indicating a possible reason for the decreased miR-497-5p levels in ER-alpha-negative breast cancer patients [28].

The prognostic role of miR-497-5p overexpression was demonstrated in nine types of cancer, though ECs were not investigated. Cancer patients with high levels of miR-497-5p have lower occurrence of lymph node metastasis and better overall survival [29]. To the best of our knowledge, only three studies have reported miR-497-5p downregulation in EC [30,31,32]. In low-risk EC patients, decreased miR-497-5p expression was observed in women with recurrence of EC compared to those without recurrence [31]. Furthermore, the analysis of RNA-Seq and miRNAseq data from The Cancer Genome Atlas (TCGA) showed that 68 differentially expressed miRNAs, including miR-497-5p, were associated with poorer survival. Decreased levels of miR-497-5p and increased expression of the *EMX1* target gene were significantly associated with advanced clinical and histopathological characteristics of ECs [32].

In this study, we first documented decreased levels of miR-497-5p in EEC, SEC and all rare EC subtypes compared to non-neoplastic endometrium. We found miR-497-5p reduced levels in high-grade ECs, both hormone-receptor-negative and only PR-negative cases, and p53-positive and highly proliferative (high Ki-67 expression) ECs compared to low-grade, hormone-receptor-positive, and p53-negative ECs and carcinomas with low proliferation. These findings correspond with our previous bioinformatic results that miR-497-5p regulates cancer cell proliferation likely through the MAPK signaling pathway [16].

Hormone receptor status has been suggested to be a relevant prognostic marker, and hormonal therapy can be used in patients with advanced or recurrent EC, particularly in low-grade EEC [33]. The presence of steroid receptors correlated with low tumor grade, as well as favorable outcome [34]. Hormonal regulation of EC is not fully understood and, to date, the evaluation of ER and PR expression is not involved in the routine care of EC patients [34,35]. A recent study indicated that EC is a hormonally driven disease and that estrogen signaling plays a key role in its tumorigenesis [36]. Estrogens bind to ER-alpha, ER-beta, and the lesser-known alternative estrogen receptor GRP30 (G-protein-coupled receptor-30), and progesterones bind to PR. These receptors are important transcription factors initiating expression of specific genes. ERs regulate cellular proliferation and differentiation, PR-A isoform modulates the anti-proliferative function of progesterone, and PR-B induces cell growth in the absence of PR-A [35,37]. Moreover, in ER-responsive cells, transcription of PR is induced by estrogen and inhibited by progesterone [38]. Changes in ER or PR expression occur during tumor progression, and expression is generally higher in primary compared to metastatic tumors [34]. In advanced ECs, reduced ER-alpha and PR-A levels were observed in epithelial glands and the stroma of EC [39]. Leslie et al. postulated that downregulation of hormone receptors resulted from constitutive phosphorylation via MAPK activation, which leads to proteasomal degradation [37]. In a previous EC study, we showed that miR-497-5p targets several genes of the MAPK signaling pathway [16]; therefore, we hypothesize that a reduction in miR-497-5p levels may allow MAPK activation and a decrease in hormonal receptor expression. This suggestion is consistent with our results that show a significantly reduced miR-497-5p expression in ECs with a loss of ER and/or PR.

Moreover, in categories highly relevant to EC diagnosis, namely in ECs with any disseminated range, such as loco-regionally or to distant organs and in ECs spreading to regions outside the uterus, decreased levels of miR-497-5p were found compared to ECs without any dissemination and metastasis, respectively. There was a discrepancy between these results and the non-significant difference in miR-497-5p expression between patients with and without metastatic lymph nodes. This could be caused by the fact that some portion of ECs may disseminate hematogenously, as was previously indicated for EECs [40].

Among other evaluated miRNAs, miR-145-5p was downregulated in EECs, but there was no significantly different expression between EECs with different grades. However, downregulated miR-145-5p was useful for discriminating rare subtypes CaSa and MC from EEC, which could indicate the involvement of miR-145-5p in any specific features of these subtypes. This hypothesis supports the results of two previous studies which reported that miR-145-5p regulates pluripotency in embryonic stem cells by targeting of pluripotency factors OCT4, SOX2, and KLF4, and suggested the role of miR-145-5p in EC differentiation [41,42]. On the other hand, previously non-significant differences in miR-143-3p and miR-424-5p between SECs and EECs were now statistically significant differences when observed in an independent group of EC patients in this study. Moreover, a downregulation in miR-143-3p was found to distinguish CaSa from EEC. This miRNA inhibits proliferation and metastasis of EC cells through *MAPK1* targeting [43], which contributes to previously presented involvement of MAPK signaling in advanced EC subtypes [16]. Another study showed that miR-424-5p inhibited EC progression by regulation of the epithelial-to-mesenchymal transition [44], but this was not applicable for distinguishing rare EC subtypes.

Due to the requests of personalized medicine, there is a visible effect in the trend of ML application that can be used to discover and identify patterns and relationships between complex datasets. ML techniques are able to effectively classify cancer types or predict patient’s future outcomes by contributing to the development of models for cancer susceptibility, recurrence, and survival prediction [14,45]. The application of ML models may decrease interobserver variability in histopathological diagnosis, which could result in less heterogeneous practice among pathologists [46]. In addition to routinely collected clinical data, histopathologic characteristics, and immunohistochemical results, multi-omics data such as genomics and transcriptomics data have been included in ML prediction models [45,47]. Among these, the ML models utilizing gene expression data can prevent potential errors in survival evaluation, help in the management of appropriate and individualized therapy and improve cancer prognosis [48]. In our study, ML was used as a diagnostic tool for prediction of EC subtypes based on clinical data combined with miRNAs analysis. The statistical results of our data show that it would be a daunting task to set a number of rules or cut-off intervals to perform cancer type classification or diagnostics. However, collecting data from 182 samples is a promising start to using ML for this purpose. ML analyses showed that the inclusion of miR-497-5p expression results in traditional clinical and immunohistochemical data slightly improved the prediction accuracy of EC diagnosis.

miRNAs regulate the expression of multiple genes and each gene could be regulated by several miRNAs; therefore, it is challenging to investigate and understand the consequences of such complex regulation processes. Regarding the molecular heterogeneity of each tumor, and also within the same subtype, a variability in miRNA expression levels could cause a problem in relation to the validation of the results. However, the interpretations of cancer prediction became be more complex and precise by involving the clinical parameters and laboratory results from many other patients into the ML algorithms, which could compensate for the variability of molecular features. Here, we found several new associations in ECs, but it is necessary to notice that the presented study has limitations for proximate clinical utility because the miRNA expression results have not yet been verified in an independent cohort of an appropriate size. miR-497-5p, like all other miRNAs, is normally expressed in human cells, and imbalances in miRNA expression can lead to the development of diseases, including cancer. From a practical point of view, the testing system for clinical utility will be based on the relative expression of tested miRNA in terms of comparing the levels in the non-neoplastic endometrium and strictly determined endometrioid and serous subtype samples, while downregulated or upregulated expression values will have diagnostic relevance.

Together, our results clearly indicate that alterations in the expression of miR-497-5p play a role in endometrial tumorigenesis, because we found the most considerable decrease in miR-497-5p levels in tumors with more aggressive behavior and those in advanced stages of disease. Downregulation of miR-497-5p was found in both endometrioid and serous carcinomas, but the significant difference in miR-497-5p levels allows the distinguishing of these two EC subtypes. Moreover, similar miR-497-5p levels as in SEC were identified in rare CaSa and CCC subtypes, which indicates a greater similarity with the serous subtype in terms of their molecular features. Using modern ML technology, we confirmed the essential role of traditional data, and miR-497-5p could be included as an additional marker for improving EC diagnosis. To verify presented results, it would be necessary to accumulate a larger independent set of samples with a random representation of different types of EC. Within this set, the evaluation of correlations identified miRNA expressions, focused predominantly on miR-497-5p, with selected indicators of biological behavior as grade, stage, lymphovascular invasion, hormone receptors status, p53 protein expression and other parameters, would be realized. These findings could be predominantly applied in the examination of biopsy specimens in the preoperative stage and in surgical and postoperative management, although these results will require further research and verifications in clinical practice.

## 4. Materials and Methods

### 4.1. Patients

In the present study, we analyzed FFPE specimens of several subtypes of endometrial carcinomas isolated from 182 patients. Briefly, 45 and 47 samples were obtained from patients with EEC G1 and EEC G3, respectively, and 44 specimens were from patients with SEC. Among the rare EC subtypes, 21 CaSas, 20 CCCs, and 5 MCs were evaluated.

The age of all patients ranged from 35 to 86 years, (mean 66.09 ± 8.50 years). Mean ages of patients with different subtypes were 64.28, 66.12, 67.78, 68.14, 67.75, and 63.4 years for EEC G1, EEC G3, SEC, CaSa, CCC, and MC groups, respectively. Samples of 20 non-neoplastic endometria from age-matched women from 53 to 79 years (mean, 65.4 ± 6.75 years) were used as controls. Subtyping of EC was evaluated according to the WHO classification of tumors [49] and histological grading presented as FIGO [50]. Clinical tumor stage was assessed according to the 2009 FIGO classification [51], and the pathological stage (tumor, lymph node status, and distant metastasis) was evaluated using the 8th TNM classification [52]. The results of the IHC analysis of hormone receptor status (ER, PR), p53 and the proliferative activity assessed by the Ki67 index were taken from biopsy reports of the included cases. Carcinoma and control samples were obtained from the tissue archives of the St. Elizabeth Cancer Institute, Bratislava, Slovakia, and prepared before 2018. The St. Elizabeth Cancer Institute Review Board approved this retrospective study and waived the consent of patients and control persons. No patient underwent preoperative radiotherapy or chemotherapy before specimen collection, and control persons had no signs or symptoms of cancer or other serious diseases. The clinical, histopathological, and immunohistochemical data of evaluated patients are summarized in Table 4.

### 4.2. miRNA Extraction and Real-Time PCR

miRNAs from FFPE endometrial tumor tissues were isolated using the miRNeasy FFPE Kit (Qiagen, Hilden, Germany) according to manufacturer’s instructions. miRNA samples of suitable purity were reversely transcribed into cDNA using the miScript II RT Kit (Qiagen) according to the manufacturer’s instructions. For real-time polymerase chain reactions of 120 patients, a Custom miScript miRNA PCR Array (CMIHS02740, Qiagen) was used for analyses of 11 selected miRNAs using the miScript SYBR Green PCR Kit (Qiagen). RT-PCR reactions were carried out in an AriaMx Real-Time PCR System (Agilent, Santa Clara, CA, USA) using the following conditions: predenaturation at 95 °C for 15 min, followed by 40 cycles at 94 °C for 15 s, 55 °C for 30 s, and 70 °C for 30 s.

### 4.3. Statistical Analysis

SPSS IBM statistics 23.0 software was used for the statistical analysis of the data. The normality of distribution was tested sing the Shapiro–Wilk test. Normally distributed variables were tested by Student’s t-test or analysis of variance (ANOVA) with Bonferroni’s or Tamhane’s corrections depending on homogeneity of variance. Mann–Whitney U or Kruskal–Wallis H tests with Dunn’s nonparametric comparisons for post hoc testing were used for non-normally distributed data. Univariate analyses for categorical variables were performed using χ2 or Fisher’s exact test. The statistically significant level was regarded as *p* < 0.05. Relative miRNA expression was represented by 2^−ΔΔCt^. For the quantification of relative gene expression, the ΔΔCt method was used [53]. Global normalization was performed using three endogenous small RNAs, namely SNORD61, SNORD72, and SNORD95. Fold change 2^−ΔΔCt^ was represented by the normalized relative gene expression in the test sample divided by the normalized gene expression in the control sample. The upregulation of miRNA expression was indicated by fold change values greater than 2 and downregulation by fold change values less than 0.5.

### 4.4. Machine Learning Analysis

**Figure 7:**
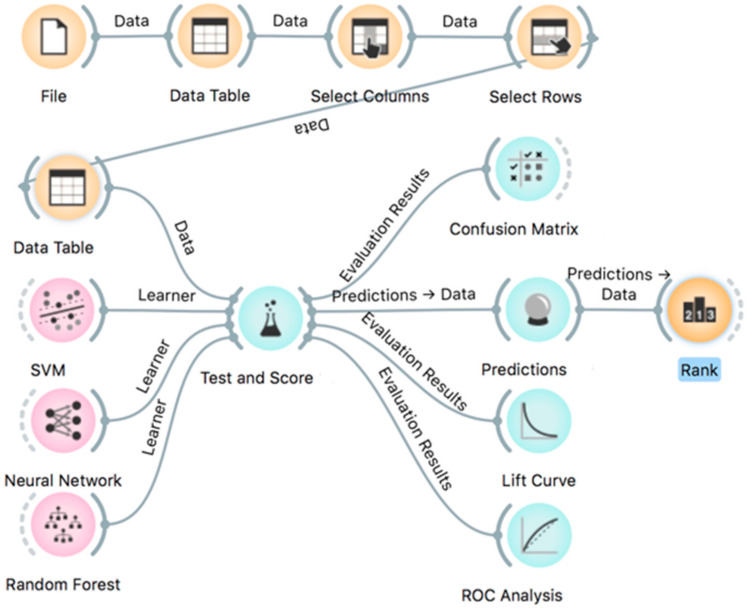
Orange workflow with selection of features and data mining tools.

ML capabilities were evaluated using Orange software version 3.26 [54], an open-source data mining tool, providing a visual approach to ML and interactive data analysis based on algorithms and libraries provided by Python [55,56]. In this study, an Orange workflow was created as shown in Figure 7. When applying an ML method, data samples constitute the basic components. Each sample was described by several input features, e.g., clinical parameters and miRNA expression results, and every feature represents different types of values, e.g., categorical, numerical, or string. SVM (support vector machine), Neural Network, and Random Forest algorithms were compared for prediction of EC subtypes considering various clinical and histopathological characteristics. Cross-validation with stratification and 10-fold sampling of input features was used for training models and for performance evaluation of prediction outcome, e.g., diagnostics of EC subtype with “Test and Score” module using Orange software. This module provides a standard performance evaluation of ML techniques (Figure 7). It calculates several performance parameters and feeds the confusion matrix, including TP (True Positive), FP (False Positive), TN (True Negative), and FN (False Negative) for actual data and predicted data. The following parameters are directly rated in the “Test and Score” module: classification accuracy (CA), precision rate, area under the ROC curve (AUC), F1 score, and recall. The parameters (features) of the prediction rating can be further evaluated by the ranking module, “Rank” (Figure 7), using several calculated scores, such as Information Gain, Gain Ratio, Gini, ANOVA (for continuous numerical features), Chi2, ReliefF. These parameters are fully described in the Orange software and they are beyond the scope of this paper. To predict the overall quality of data mining, the Orange software visual tool Lift Curve module was used. The lift curve was obtained by plotting the true-positive predictions (TP rate) against the actual total number of positive instances (P rate) as a measure of the proficiency of the classifier algorithm.

## Figures and Tables

**Table 1:** Verification of expression differences in selected miRNAs when comparing different subtypes of endometrial carcinomas.

**Downregulated**	**EEC G1 vs. N**			**EEC G3 vs. N**			**EEC G3 vs. G1**		
**miRNA**	**FC**	***p*-Value**	**95% CI**	**FC**	***p*-Value**	**95% CI**	**FC**	***p*-Value**	**95% CI**
let-7c-5p	0.593	0.005	(0.378,0.853)	0.482	<0.001	(0.334,0.665)	0.812	0.359	(0.525,1.312)
miR-125b-5p	0.199	<0.001	(0.101,0.314)	0.186	<0.001	(0.101,0.286)	0.932	0.83	(0.463,1.966)
miR-23b-3p	0.453	<0.001	(0.243,0.693)	0.351	<0.001	(0.154,0.572)	0.776	0.212	(0.321,1.626)
miR-99a-5p	0.176	<0.001	(0.083,0.288)	0.159	<0.001	(0.075,0.259)	0.901	0.777	(0.390,2.083)
miR-145-5p	0.230	<0.001	(0.099,0.390)	0.262	<0.001	(0.034, 0.523)	1.138	0.808	(0.148,3.143)
	**EEC vs. N**			**SEC vs. N**			**SEC vs EEC**		
	**FC**	***p*-Value**	**95% CI**	**FC**	***p*-Value**	**95% CI**	**FC**	***p*-Value**	**95% CI**
let-7g-5p	1.059	0.700	(0.773,1.397)	0.518	<0.001	(0.371,0.692)	0.490	<0.001	(0.339,0.703)
miR-195-5p	0.423	<0.001	(0.333,0.534)	0.409	0.0005	(0.139,0.699)	0.966	0.917	(0.335,1.658)
miR-34a-5p	0.658	0.003	(0.515,0.846)	0.480	0.001	(0.242,0.752)	0.729	0.172	(0.373,1.126)
miR-497-5p	0.256	<0.001	(0.180,0.351)	0.081	<0.001	(0.054,0.115)	0.318	<0.001	(0.204,0.480)
miR-143-3p	0.185	<0.001	(0.112,0.287)	0.089	<0.001	(0.040,0.152)	0.481	0.023	(0.212,0.896)
miR-424-5p	0.263	<0.001	(0.109,0.442)	0.074	<0.001	(0.0450,0.110)	0.281	0.018	(0.148,0.699)

Abbreviations: EEC, endometrioid endometrial carcinoma; SEC, serous endometrial carcinoma; G1 and G3, grade 1 and grade 3; N, non-neoplastic endometrium; FC, fold change; CI, confidence interval.

**Table 2:** Rare subtypes of endometrial carcinoma: expression of miRNAs selected for discrimination of endometrioid endometrial carcinomas according to different grades.

EC Subypes	let-7c-5p		miR-125b-5p		miR-23b-3p		miR-99a-5p		miR-145-5p	
	FC	*p*-Value	FC	*p*-Value	FC	*p*-Value	FC	*p*-Value	FC	*p*-Value
CaSa vs. N	0.726	0.187	0.156	<0.001	0.229	<0.001	0.121	<0.001	0.052	<0.001
CCC vs. N	0.386	<0.001	0.160	<0.001	0.301	<0.001	0.104	<0.001	0.136	<0.001
MC vs. N	0.635	0.232	0.087	<0.001	0.353	<0.001	0.076	<0.001	0.112	<0.001
CaSa vs. EEC G1	1.223	0.526	0.782	0.432	0.505	0.048	0.681	0.279	0.044	<0.001
CaSa vs. EEC G3	1.507	0.215	0.839	0.543	0.651	0.252	0.756	0.415	0.071	<0.001
CCC vs. EEC G1	0.650	0.151	0.800	0.574	0.664	0.247	0.589	0.249	0.663	0.164
CCC vs. EEC G3	0.802	0.431	0.859	0.692	0.856	0.690	0.654	0.355	1.069	0.801
MC vs. EEC G1	1.070	0.884	0.434	0.128	0.779	0.564	0.431	0.090	0.094	<0.001
MC vs. EEC G3	1.319	0.585	0.466	0.156	1.005	0.992	0.478	0.134	0.152	<0.001
CaSa vs. CCC	1.880	0.109	0.977	0.950	0.761	0.432	1.155	0.738	0.385	0.168
CaSa vs. MC	1.143	0.777	1.801	0.265	0.648	0.421	1.581	0.305	0.467	0.402
CCC vs. MC	0.608	0.399	1.844	0.328	0.851	0.745	1.369	0.607	1.214	0.785

Abbreviations: CaSa, carcinosarcoma; CCC, clear cell carcinoma; MC, mucinous carcinoma; N, non-neoplastic endometrium; EEC, endometrioid endometrial carcinoma; G1 and G3, grade 1 and grade 3; FC, fold change. Confidence interval values are included in Table S2.

**Table 3:** Rare subtypes of endometrial carcinoma: expression of miRNAs selected for discrimination of endometrioid and serous subtypes.

EC Subtypes	let-7g-5p		miR-195-5p		miR-34a-5p		miR-497-5p		miR-143-3p		miR-424-5p	
	FC	*p*-Value	FC	*p*-Value	FC	*p*-Value	FC	*p*-Value	FC	*p*-Value	FC	*p*-Value
CaSa vs. N	0.593	0.005	0.239	<0.001	0.431	0.002	0.120	<0.001	0.054	<0.001	0.186	<0.001
CCC vs. N	0.912	0.693	0.341	<0.001	0.454	<0.001	0.116	<0.001	0.103	<0.001	0.098	<0.001
MC vs. N	2.108	0.506	0.547	0.023	0.529	0.008	0.173	<0.001	0.057	<0.001	0.131	<0.001
CaSa vs. EEC	0.560	0.008	0.565	0.0001	0.655	0.151	0.467	0.005	0.293	<0.001	0.709	0.428
CaSa vs. SEC	1.144	0.567	0.585	0.222	0.899	0.793	1.470	0.275	0.608	0.176	2.522	0.073
CCC vs. EEC	0.861	0.552	0.806	0.322	0.689	0.127	0.454	0.008	0.557	0.096	0.372	0.041
CCC vs. SEC	1.759	0.084	0.835	0.659	0.945	0.874	1.428	0.389	1.157	0.743	1.325	0.316
MC vs. EEC	1.992	0.528	1.293	0.421	0.804	0.335	0.675	0.101	0.308	0.034	0.499	0.168
MC vs. SEC	4.067	0.354	1.339	0.479	1.103	0.763	2.123	0.048	0.639	0.517	1.777	0.348
CaSa vs. CCC	0.650	0.185	0.700	0.194	0.951	0.905	1.029	0.946	0.526	0.184	1.904	0.161
CaSa vs. MC	0.281	0.375	0.437	0.085	0.815	0.716	0.693	0.207	0.951	0.949	1.420	0.495
CCC vs. MC	0.432	0.477	0.623	0.225	0.857	0.648	0.673	0.278	1.810	0.407	0.746	0.580

Abbreviations: CaSa, carcinosarcoma; CCC, clear cell carcinoma; MC, mucinous carcinoma; N, non-neoplastic endometrium; EEC, endometrioid endometrial carcinoma; SEC, serous endometrial carcinoma; FC, fold change. Confidence interval values are included in Table S3.

**Table 4:** Clinical characteristics of endometrial cancer patients.

Variables		N	%
**All patients**		182	100
**Histological subtype**	EEC	92	50.55
	SEC	44	24.18
	CaSa	21	11.54
	CCC	20	10.99
	MC	5	2.75
**Grade**	EEC grade 1	45	48.91
	EEC grade 3	47	51.09
	Low *	50	27.47
	High **	132	72.53
**FIGO stage**	IA	70	38.46
	IB	24	13.19
	II	22	12.09
	IIIA	6	3.3
	IIIB	1	0.55
	IIIC1	18	9.89
	IIIC2	9	4.95
	IVB	32	17.58
**T (tumor)**	T1a	82	45.05
	T1b	33	18.13
	T2	34	18.68
	T3a	28	15.38
	T3b	5	2.75
**LN status**	N0	98	70.5
	N1	31	22.3
	N2	10	7.19
**Metastatic status**	Negative	85	72.65
	Positive	32	27.35
**HR status**	ER−/PR−	51	28.02
	ER+/PR+	111	60.99
	ER+/PR−	18	9.89
	ER−/PR+	2	1.1
**ER status**	Positive	129	70.88
	Negative	53	29.12
**PR status**	Positive	113	62.09
	Negative	69	37.91
**p53 status**	Negative	111	60.99
	Positive	71	39.01
**Ki-67 proliferative index**	Low	69	37.91
	High	113	62.09

* EEC G1 and MC; ** EEC G3, SEC, CaSa and CCC. Abbreviations: EEC, endometrioid endometrial carcinoma; SEC, serous endometrial carcinoma; CaSa, carcinosarcoma; CCC, clear cell carcinoma, MC, mucinous carcinoma; HR status, status of hormonal receptors; LN status, status of lymph nodes; N0, no regional lymph node metastasis; N1, regional lymph node metastasis to pelvic lymph nodes; N2, regional lymph node metastasis to paraaortic lymph nodes with or without metastasis to pelvic lymph nodes; metastatic status negative, no metastases; positive, one or more metastasis; ER/PR status, positive in cases with ˃1% of positively responding cells; p53 status, negative cases presented <90% of responsive cells and positive had ≥90% of responsive cells or showed complete absence of p53 in all affected cells; Ki-67 proliferative index, low and high represented ≤50% and ˃50% of responsive cells, respectively.

**Table 5:** Summarized confusion matrix results for SVM (support vector machine) and selected groups of clinical and histopathological characteristics of endometrial carcinoma.

Tumor Characteristics	ER, PR, Ki67, P53		ER, PR, Ki67, P53, T, P53, T, FIGO, Grade		ER%, PR%, Ki67%, Age		ER%, PR%, Ki67%, P53, Age, T, FIGO, Grade	
	Prediction		Prediction		Prediction		Prediction	
	% (95% ±ΔCI)		% (95% ±ΔCI)		% (95% ±ΔCI)		% (95% ±ΔCI)	
miRNA -497-5p	without	with	without	with	without	with	without	with
EEC G1	86.7 (8.3)	88.9 (8.0)	91.1 (6.31)	93.3 (5.99)	75.6 (10.2)	77.8 (10.4)	88.9 (6.7)	93.3 (6.0)
EEC G3	66.0 (11.3)	72.3 (10.9)	61.7 (11.8)	59.6 (12.2)	46.8 (12.6)	51.1 (12.1)	59.6 (12.3)	61.7 (12.1)
SEC	59.1 (11.9)	65.9 (11.6)	59.1 (12.0)	52.3 (12.7)	63.6 (12.9)	63.6 (12.5)	47.7 (12.6)	52.3 (12.3)
CaSa	47.6 (14.2)	28.6 (15.2)	71.4 (13.1)	57.1 (13.4)	42.9 (14.0)	47.6 (13.6)	61.9 (12.4)	61.9 (13.1)
CCC	55.0 (7.1)	50.0 (7.1)	50.0 (11.3)	45.0 (11.7)	55.0 (10.4)	45.0 (11.3)	50.0 (11.7)	50.0 (11.3)
MC	0.0 (20.0)	0.0 (20.8)	0.0 (14.2)	0.0 (15.0)	0.0 (14.2)	0.0 (15.7)	20.0 (15.7)	20.0 (14.2)

Abbreviations: EEC, endometrioid endometrial carcinoma; SEC, serous endometrial carcinoma; G1 and G3, grade 1 and grade 3; CaSa, carcinosarcoma; CCC, clear cell carcinoma; MC, mucinous carcinoma; ER, estrogen receptor; PR, progesterone receptor; Ki-67 proliferative index; T, tumor classification according to TNM Classification of Malignant Tumors; CI, confidence interval.

## Supplementary Materials

The following are available online at https://www.mdpi.com/1422-0067/22/1/127/s1.

## Abbreviations

EC	endometrial cancer
EEC	endometrioid endometrial cancer
G	grade
SEC	serous endometrial cancer
CCC	clear cell carcinoma
CaSa	carcinosarcoma
MC	mucinous carcinoma
FIGO	International Federation of Gynecology and Obstetrics
LN	lymph node
LVSI	lymphovascular space invasion
IQR	interquartile range
N	non-neoplastic endometrium
MTS	distant metastases
miRNA	microRNAs
ML	machine learning
SVM	support vector machine
CA	classification accuracy
AUC	area under ROC curve
F1	F-score
PR	progesterone receptor
ER	estrogen receptor
ERR	estrogen-related receptor
GRP30	G-protein-coupled receptor-30
FFPE	formalin-fixed paraffin embedded
MAPK	mitogen-activated protein kinases
EMT	epithelial–mesenchymal transition
TP	true-positive predictions
TNM	Classification of Malignant Tumors
