# Supplementary material for: miR-497-5p Decreased Expression Associated with High-Risk Endometrial Cancer
Source: Int J Mol Sci. 2020 Dec 24;22(1):127. doi: 10.3390/ijms22010127 (PMC7795869; doi:10.3390/ijms22010127)
Supplement: Supplementary file 1 [file ijms-22-00127-s001.pdf]

Supplementary Material

Table S1. Expressions of selected miRNAs when comparing different subtypes of endometrial carcinomas reported in previous study in 62 patients [16].

Downregulated miRNA	EEC G1 vs. N		EEC G3 vs. N		EEC G3 vs. G1	
	FC	*P*-value	FC	*P*-value	FC	*P*-value
let-7c-5p	0.353	< 0.001	0.171	< 0.001	0.487	0.003
miR-125b-5p	0.263	< 0.001	0.123	< 0.001	0.469	0.012
miR-23b-3p	0.353	< 0.001	0.145	< 0.001	0.404	0.002
miR-99a-5p	0.251	< 0.001	0.120	< 0.001	0.480	0.011
miR-145-5p	0.265	< 0.001	0.109	< 0.001	0.414	0.056
	EEC vs. N		SEC vs. N		SEC vs EEC	
	FC	*P*-value	FC	*P*-value	FC	*P*-value
let-7g-5p	0.510	< 0.001	0.211	< 0.001	0.413	0.005
miR-195-5p	0.394	< 0.001	0.190	< 0.001	0.482	0.002
miR-34a-5p	0.721	0.040	0.264	< 0.001	0.367	0.001
miR-497-5p	0.339	< 0.001	0.085	< 0.001	0.250	< 0.001
miR-143 -3p	0.194	< 0.001	0.071	< 0.001	0.363	0.185
miR-424-5p	0.055	< 0.001	0.027	< 0.001	0.492	0.267

Abbreviations: EEC, endometrioid endometrial carcinoma; SEC, serous endometrial carcinoma; G1 and G3, grade 1 and grade 3; N, non-neoplastic endometrium; FC, fold change.

Table S2. Rare subtypes of endometrial carcinoma: expression of miRNAs selected for discrimination of endometrioid endometrial carcinomas according to different grades including the confidence interval values.

EC subypes	let-7c-5p			miR-125b-5p			miR-23b-3p			miR-99a-5p			miR-145-5p		
	FC	*P*-value	95% CI	FC	*P*-value	95% CI	FC	*P*-value	95% CI	FC	*P*-value	95% CI	FC	*P*-value	95% CI
CaSa vs. N	0.726	0.187	(0.355,1.151)	0.156	< 0.001	(0.099,0.226)	0.229	< 0.001	(0.144,0.329)	0.121	< 0.001	(0.072,0.181)	0.052	< 0.001	(0.031,0.080)
CCC vs. N	0.386	< 0.001	(0.181,0.620)	0.16	< 0.001	(0.055,0.277)	0.301	< 0.001	(0.135,0.486)	0.104	< 0.001	(0.019,0.199)	0.136	< 0.001	(0.019,0.270)
MC vs. N	0.635	0.232	(0.112,1.208)	0.087	< 0.001	(-0.017,0.198)	0.353	< 0.001	(0.078,0.652)	0.076	< 0.001	(0.007,0.153)	0.112	< 0.001	(-0.019,0.258)
CaSa vs. EEC G1	1.223	0.526	(0.608,2.163)	0.782	0.432	(0.380,1.176)	0.505	0.048	(0.285,0.984)	0.681	0.279	(0.365,1.507)	0.358	< 0.001	(0.025,0.077)
CaSa vs. EEC G3	1.507	0.215	(0.764,2.501)	0.839	0.543	(0.480,1.609)	0.651	0.252	(0.350,1.538)	0.756	0.415	(0.406,1.671)	0.071	< 0.001	(0.042,0.108)
CCC vs. EEC G1	0.65	0.151	(0.296,1.178)	0.8	0.574	(0.264,1.826)	0.664	0.247	(0.282,1.387)	0.589	0.249	(0.107,1.515)	0.663	0.164	(0.016,0.249)
CCC vs. EEC G3	0.802	0.431	(0.371,1.366)	0.859	0.692	(0.286,1.852)	0.856	0.69	(0.352,2.130)	0.654	0.355	(0.119,1.680)	1.069	0.801	(0.026,0.364)
MC vs. EEC G1	1.07	0.884	(0.243,2.620)	0.434	0.128	(-0.084,1.229)	0.779	0.564	(0.175,1.801)	0.431	0.09	(0.044,1.156)	0.094	< 0.001	(-0.015,0.235)
MC vs. EEC G3	1.319	0.585	(0.232,2.406)	0.466	0.156	(-0.089,1.257)	1.005	0.992	(0.223,2.731)	0.478	0.134	(0.049,1.280)	0.152	< 0.001	(-0.024,0.347)
CaSa vs. CCC	1.88	0.109	(0.839,4.345)	0.977	0.95	(0.505,2.904)	0.761	0.432	(0.412,1.749)	1.155	0.738	(0.533,6.365)	0.385	0.168	(0.283,1.686)
CaSa vs. MC	1.143	0.777	(0.450,6.571)	1.801	0.265	**	0.648	0.421	(0.312,2.923)	1.581	0.305	(0.695,16.610)	0.467	0.402	**
CCC vs. MC	0.608	0.399	(0.231,3.540)	1.844	0.328	**	0.851	0.745	(0.318,3.963)	1.369	0.607	(0.211,15.084)	1.214	0.785	**

** confidence interval of the denominator includes zero

Abbreviations: CaSa, carcinosarcoma; CCC, clear cell carcinoma; MC, mucinous carcinoma; N, non-neoplastic endometrium; EEC, endometrioid endometrial carcinoma; G1 and G3, grade 1 and grade 3; FC, fold change; CI, confidence interval.

Table S3. Rare subtypes of endometrial carcinoma: expression of miRNAs selected for discrimination of endometrioid and serous subtypes including the confidence interval values.

EC subtypes	let-7g-5p			miR-195-5p			miR-34a-5p			miR-497-5p			miR-143 -3p			miR-424-5p		
	FC	*P*-value	95% CI	FC	*P*-value	95% CI	FC	*P*-value	95% CI	FC	*P*-value	95% CI	FC	*P*-value	95% CI	FC	*P*-value	95% CI
CaSa vs. N	0.593	0.005	(0.36,0.85)	0.239	< 0.001	(0.18,0.30)	0.431	0.002	(0.13,0.75)	0.12	< 0.001	(0.04,0.23)	0.054	< 0.001	(0.03,0.08)	0.186	< 0.001	(0.06,0.32)
CCC vs. N	0.912	0.693	(0.48,1.38)	0.341	< 0.001	(0.19,0.50)	0.454	< 0.001	(0.21,0.72)	0.116	< 0.001	(0.03,0.17)	0.103	< 0.001	(0.03,0.19)	0.098	< 0.001	(0.05,0.14)
MC vs. N	2.108	0.506	(-1.03,5.36)	0.547	0.023	(0.26,0.86)	0.529	0.008	(0.29,0.80)	0.173	< 0.001	(0.08,0.31)	0.057	< 0.001	(-0.02,0.15)	0.131	< 0.001	(0.02,0.25)
CaSa vs. EEC	0.56	0.008	(0.33,0.85)	0.565	0.0001	(0.42,0.74)	0.655	0.151	(0.21,1.12)	0.467	0.005	(0.21,0.79)	0.293	< 0.001	(0.18,0.48)	0.709	0.428	(0.24,1.89)
CaSa vs. SEC	1.144	0.567	(0.66,1.84)	0.585	0.222	(0.32,2.01)	0.899	0.793	(0.27,2.19)	1.47	0.275	(0.65,2.61)	0.608	0.176	(0.34,1.49)	2.522	0.073	(0.89,5.09)
CCC vs. EEC	0.861	0.552	(0.45,1.37)	0.806	0.322	(0.45,1.20)	0.689	0.127	(0.32,1.08)	0.454	0.008	(0.15,0.81)	0.557	0.096	(0.17,1.10)	0.372	0.041	(0.18,0.93)
CCC vs. SEC	1.759	0.084	(0.90,2.95)	0.835	0.659	(0.38,2.96)	0.945	0.874	(0.40, 2.20)	1.428	0.389	(0.48,2.69)	1.157	0.743	(0.34,3.16)	1.325	0.316	(0.72,2.42)
MC vs. EEC	1.992	0.528	(-0.89,5.13)	1.293	0.421	(0.64,2.02)	0.804	0.335	(0.45,1.19)	0.675	0.101	(0.39,1.06)	0.308	0.034	(-0.13,0.84)	0.499	0.168	(0.09,1.42)
MC vs. SEC	4.067	0.354	(-2.02,10.98)	1.339	0.479	(0.54,5.07)	1.103	0.763	(0.54,2.54)	2.123	0.048	(1.19,3.54)	0.639	0.517	(-0.32,2.30)	1.777	0.348	(0.28,3.96)
CaSa vs. CCC	0.65	0.185	(0.35,1.29)	0.7	0.194	(0.45,1.26)	0.951	0.905	(0.29,2.34)	1.029	0.946	(0.41,3.18)	0.526	0.184	(0.28,1.68)	1.904	0.161	(0.67,3.85)
CaSa vs. MC	0.281	0.375	**	0.437	0.085	(0.27,0.91)	0.815	0.716	(0.24,1.76)	0.693	0.207	(0.29,1.35)	0.951	0.949	**	1.42	0.495	(0.43,9.00)
CCC vs. MC	0.432	0.477	**	0.623	0.225	(0.30,1.38)	0.857	0.648	(0.36,1.74)	0.673	0.278	(0.21,1.38)	1.81	0.407	**	0.746	0.58	(0.33,4.67)

** confidence interval of the denominator includes zero

Abbreviations: CaSa, carcinosarcoma; CCC, clear cell carcinoma; MC, mucinous carcinoma; N, non-neoplastic endometrium; EEC, endometrioid endometrial carcinoma; SEC, serous endometrial carcinoma, FC, fold change; CI, confidence interval.

Table S4. Comparative evaluation of the performance for three classification methods on sub-samples of EEC and SEC subtypes.

Method	AUC	Accuracy	F1 score	Precision	Recall
SVM	0.890	0.626	0.601	0.584	0.626
Neural Network	0.900	0.687	0.675	0.669	0.687
Random Forest	0.890	0.714	0.700	0.692	0.714

Abbreviations: SVM, support vector machines; AUC, area under ROC curve.

Table S5. Prediction comparison of SVM and Neural Network methods trained by bootstrap with replicable and stratifying sampling (options in Orange software) for EECG1, EECG3 and SEC subtypes.

Sampler type	Prediction in % All ECs	Prediction in % EEC G3	False positive in % EEC G3	False negative in % EEC G3	EEC G1 count	EEC G3 count	SEC count	Total samples
SVM (35% remain)	65.96	43.75	8.51	19.15	16	16	15	47
Neural Network (35% remain)	61.70	50.00	17.02	17.02	16	16	15	47
SVM (bootstrap remain)	69.09	35.29	9.09	20.00	22	17	16	55
Neural Network (bootstrap remain)	58.18	35.29	21.82	20.00	22	17	16	55

Abbreviations: EEC, endometrioid endometrial carcinoma; G1 and G3, grade 1 and grade 3; SVM, support vector machines.

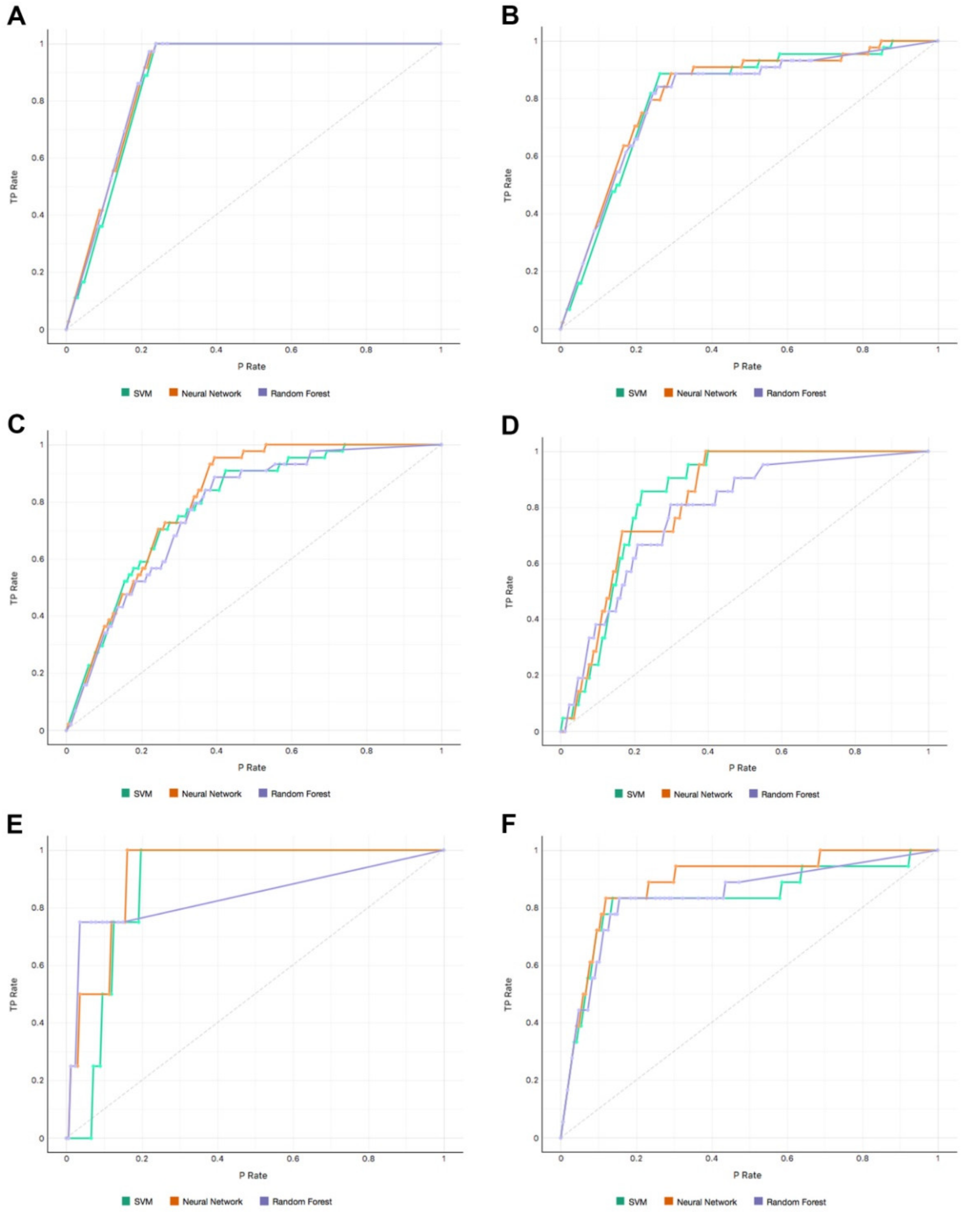

Figure S1. The lift curves for the data mining analyses.

Input features ER%, PR%, Ki67%, p53, Grade, and miR-497-5p of lift curves for target subtype endometrioid endometrial carcinoma grade 1 (EEC G1) (**A**), endometrioid endometrial carcinoma grade 3 (EEC G3) (**B**), serous endometrial carcinoma (SEC) (**C**), carcinosarcoma (CaSa) (**D**), mucinous endometrial carcinoma (MC) (**E**), and clear cell carcinoma (CCC) (**F**).
